# Supplementary material for: Postmortem Cardiopulmonary Pathology in Patients with COVID-19 Infection: Single-Center Report of 12 Autopsies from Lausanne, Switzerland
Source: Diagnostics (Basel). 2021 Jul 28;11(8):1357. doi: 10.3390/diagnostics11081357 (PMC8393761; doi:10.3390/diagnostics11081357)
Supplement: Supplementary file 1 [file diagnostics-11-01357-s001.zip › diagnostics-1259093-supplementary.pdf]

Supplementary Materials:

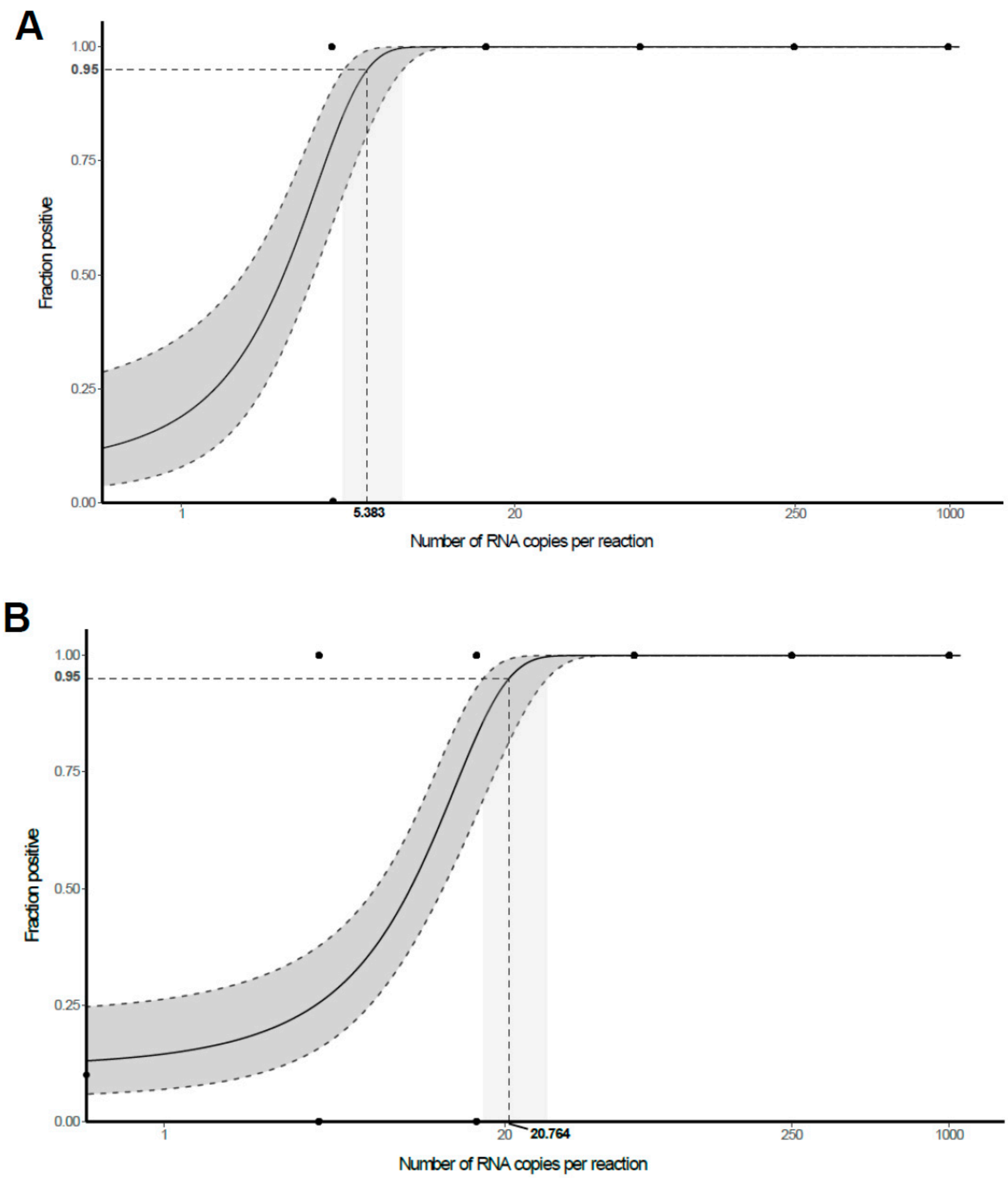

FigureS1. DeterminationoflimitsofdetectionLoD)basedon*invitro*transcribedviralRNAofEgeneandRdRp.Pro-bitanalysisrevealedaLoDof5.383(95%CI:4.365-7.409)RNAcopiesperreactionat95%probabilityforEgene-assay(A)and20.764(95%CI:16.565-29.067)forRdRpassay(B).

Supplementary Table S1. Clinical details on the patient cohort.

Case	Gender, age (years)	BMI (kg/m2)	Duration of symptoms, [mechanical ventilation] (days)	Medication		Comorbidities and risk factors		
				**COVID-19 related treatments (antiviral, antibiotics, hydroxychloroquin, corticosteroids)**	**Other relevant medication (anticoagulation and/or interfering with RAAS*)**	**Cardio-vascular**	**Pulmonary**	**others**
1	F, 72	31.6	6 [-]	Lopinavir/ritonavir (Kaletra)<br>Remdesevir	Lisinopril§	-cardiomyopathy (arrhythmogenic, hypertensive and toxic due to chemotherapy)<br>-systemic hypertension	-sleep apnea syndrome	-follicular lymphoma grade I-II (in remission)<br>-lymphopenia (0.23G/L)<br>-hypothyroidism (substitution therapy)<br>-mixed anxiety-depressive disorder (treated with Citalopram)
2	F, 72	22.1	8 [-]	Co-amoxicillin<br>Piperacillin–Tazobactam	Furosemide<br>Apixaban*	-systemic hypertension with peripheral arterial disease	-COPD, not staged<br>-former smoker (58py), stopped 3 years ago	-chronic renal insufficiency, not staged<br>-bipolar disorder, under lithium treatment<br>-substituted hypothyroidism
3	M, 96	25.3	8 [-]	Co-amoxicillin<br>Hydroxychloroquin	Apixaban*	-	-h/o community acquired pneumonia 1 year ago<br>-h/o pulmonary embolism (right middle and	-chronic renal insufficiency KDIGO G3bAx<br>-severe scoliosis<br>-femoral head prosthesis since 2 years<br>-h/o TURP

							inferior lobe) 1 year ago	
4	M, 86	24.2	10 [3]	Atazanavir (Reyataz)<br>Co-amoxicillin	Calciparine: unfractioned heparin*	-DM type II<br>-dyslipidemia	-	
5	F, 74	33.3	11 [5]	Levofloxacin<br>Piperacillin<br>Tazobactam<br>Atanazavir<br>Hydroxychloroquin<br>Remdesevir	Clexane: low molecular weight heparin*	-arrhythmogenic cardiomyopathy (pacemaker)<br>-systemic hypertension	-COPD stage III<br>-asthma<br>-sleep apnea syndrome	-mixed anxiety-depressive disorder (treated)<br>-hiatal hernia<br>-substituted hypothyroidism following subtotal thyroidectomy for multinodular goiter
6	F, 71	28.7	14 [-]	Hydroxychloroquin<br>Ceftriaxone		-ischemic cardiomyopathy<br>-h/o myocardial infarction (not dated)<br>-h/o myocarditis 8 years ago		-regenerative normocytic normochromic anemia<br>-acute confusional state (hypo and hyperactive) of multifactorial origin<br>-gait and balance instability<br>-mixed anxiety-depressive disorder<br>-moderate mental retardation<br>-h/o hysterectomy (not dated)<br>-h/o urinary sepsis with renal insufficiency (KDIGO stage 3) 1 year ago
7	M, 35	21	15 [2]	Piperacillin/<br>Tazobactam		-	-	-Tetraplegia (due to a road accident 13 years ago)
8	M, 79	28	16 [4]	Amoxicillin-clavulanic acid<br>Tazobactam<br>Hydroxychloroquin<br>Atazanavir	Salbutamol<br>Anticoagulants (not specified) *	-DM type II<br>-dyslipidemia<br>-AVB 1st degree and LAFB	-COPD stage II<br>-asthma	-prostate cancer (autopsy finding)<br>-pancreatic neuroendocrine tumor (autopsy finding)<br>-ankylosig spondylarthritis

								-h/o cerebellar ischemic stroke (9 years ago)
9	M, 75	24.7	17 [5]	Amoxicillin-clavulanic acid<br>Flucloxacillin<br>Rifampicin<br>Gentamicin<br>Meropenem<br>Atazanavir	Acenocoumarol*<br>Spironolactone§<br>Perindopril§	-DM type II<br>-hypertrophic and valvular cardiomyopathy<br>-h/o aortic valve replacement<br>-pacemaker due to complete heart block<br>-systemic hypertension	-former smoker	-metatarso-phalangeal osteoarthritis (left hallux) due to diabetic foot ulcer with secondary bacteriemia (methicillin-susceptible Staphylococcus aureus and Proteus mirabilis) (1 month before death)
10	F, 73	26	21 [14]	Hydrochloroquin<br>Lopinavir/ritonavir<br>Piperacillin-tazobactam<br>Ceftriaxone	Clexane: low molecular weight heparin*	-systemic hypertension		-lung cancer (autopsy finding)<br>-paranoid schizophrenia (institutionalized patient)<br>-h/o breast carcinoma (8 years ago)<br>-malnutrition
11	M, 60	25.1	18# [17]	Vancomycin Rifampicin<br>Imipenem	Lysis therapy (suspicion for pulmonary embolism) *	-DM type II<br>-systemic hypertension with peripheral arterial disease<br>-ischemic cardiomyopathy (h/o coronary stenting 1 year ago)	-COPD with asthma component<br>-active smoking (80py)	-alcoholism<br>-prostate cancer (autopsy finding)
12	M, 69	23	38# [22]	Hydroxycholorquin<br>Amoxicillin-clavulanic acid	Clopidogrel*, ASA*, UFH*<br>Levosimendan	-dilated cardiomyopathy		

				Azithromycin<br>Piperacillin-tazobactam<br>Cefepime<br>Meropenem<br>Vancomycin		(mixed: ischemic, hypertensive, genetic)<br>-h/o coronary stenting<br>-automatic defibrillator (since 9 years)<br>-systemic hypertension<br>-dyslipidemia		

#Duration of symptoms unknown; the number of days provided is the duration of hospitalization.
*Anticoagulants
§agents interfering with renin–angiotensin–aldosterone system (RAAS), directly or indirectly: ACE-Inhibitors, AT1-Inhibitors (ARBs), renin-inhibitors, aldosterone-antagonists
ASA, acetylsalicylic acid; AVB, atrioventricular block; COPD, chronic obstructive pulmonary disease; DM, diabetes mellitus; h/o, history of; KDIGO, "Kidney Disease: Improving Global Outcomes"; py, pack years; TURP, transurethral resection of the prostate; UFH, Unfractionated heparin;

Supplementary Table S2. Postmortem cardio-pulmonary and other significant findings in the patient cohort.

Case	Number of FFPE blocks		Lung findings	Heart findings	Malignant neoplastic disease	Other significant findings
	Lung	Heart				
1	27	16	-weight: 960g<br>-DAD exudative phase<br>-pleural effusion: absent	weight 480g; hypertrophy, patchy fibrosis	Follicular lymphoma, in complete remission	Spleen: atrophy and necrosis (shock related) Liver: low-grade centrilobular, macrovesicular steatosis; Pancreas with low-grade chronic fibrosis
2	14	6	-weight: 1200g<br>-DAD exudative phase<br>-acute bronchopneumonia, upper lobe predominant, focally with aspirate<br>-pleural effusion: 60ml/60ml	weight 420g; biventricular hypertrophy; CAD with stenosis up to 70%; fibrotic scar left ventricle (0.7cm);		signs of shock in kidney and liver; liver: lobular inflammation and necrosis, steatosis grade 1 and low-grade fibrosis (METAVIR F1); moderate systemic arteriosclerosis
3	28	7	-weight: 1500g<br>-DAD late exudative phase<br>-acute bronchopneumonia in all lobes	weight 400g; hypertrophy; CAD with stenosis up to 30%; patchy fibrosis		Liver: low-grade macrovesicular steatosis (grade 1) and low-grade fibrosis (METAVIR F1); severe systemic arteriosclerosis;

			-pleural effusion: 40ml/60ml			
4	28	4	-weight: 2390g<br>-DAD, late exudative/early proliferative phase<br>-focal interstitial and vascular amyloidosis<br>-pleural effusion: 240ml/180ml	weight 420g; hypertrophy, CAD with stenosis up to 50%; low-grade diffuse interstitial fibrosis		Diabetic and hypertensive nephropathy; moderate systemic arteriosclerosis; chronic gastritis.
5	43	4	-weight: 1970g<br>-DAD exudative phase<br>-acute bronchopneumonia in all lobes<br>-pleural effusion: 120ml/120ml	weight 330g; hypertrophy; pacemaker in place.		Kidney stones; moderate systemic arteriosclerosis; Liver: macrovesicular steatosis grade 2; gallbladder stones;
6	18	8	-weight: 1870g<br>-DAD late exudative phase/focally proliferative phase<br>-acute bronchopneumonia in all lobes<br>-pleural effusion: absent	weight 410g; status post myocardial infarction with a 1.5 cm scar (apical posterior left ventricle)		Hepatomegaly with macrovesicular steatosis grade 3; hypertensive nephropathy and signs of shock in the kidneys; severe systemic arteriosclerosis
7	17	6	-weight 1630g<br>-acute and necrotizing aspiration bronchopneumonia in all lobes<br>-No DAD.<br>(clinical/radiological picture: aspiration pneumonia)<br>-pleural effusion: absent	weight 360g		none
8	23	6	-weight: 2060g<br>-DAD proliferative phase<br>-bilateral peripheral emboli with focal, small hemorrhagic infarction<br>-pleural effusion: 360ml/40ml	weight 390g; concentric right ventricular hypertrophy; focal fibrosis in one left papillary muscle;	-Adenocarcinoma of the prostate (aT2c aN0 aM0; Gleason grade 3+3=6; Grade group 1);<br>-Pancreatic neuroendocrine tumor (NET G1; 0.7cm diameter)	signs of shock in the kidneys; light steatotic hepatomegaly (grade 1); splenomegaly; moderate systemic arteriosclerosis;

9	15	8	-weight 2560g<br>-DAD late exudative phase, upper lobe predominant<br>-pleural effusion: 480ml/180ml	weight 870g; biventricular hypertrophy; with patchy fibrosis; mechanical aortic valve; pacemaker in place		signs of shock in kidney and liver; Hepatomegaly with steatosis grade 2; moderate systemic arteriosclerosis
10	20	5	-weight: 1930g<br>AFOP in all lobes<br>-squamous cell carcinoma left lower lobe, central<br>-mucostasis<br>(microbiology: *proteus mirabilis*)<br>-pleural effusion: absent	weight 510g ; hypertrophy, CAD with stenosis up to 30%;	Squamous cell carcinoma left lower lobe, central, diameter 6cm (aT3 aN1 aM0)	Hepatomegaly, Splenomegaly; severe systemic arteriosclerosis
11	26	5	-weight: 1860g<br>-DAD proliferative phase<br>-aspergillosis in the upper left lobe with bronchial ulceration and associated focal peribronchial abscessing bronchopneumonia<br>-pleural effusion: absent	weight 470g, hypertrophy; CAD with stenosis up to 80%	-Adenocarcinoma of the prostate (aT2c aN0 aM0; Gleason grade 3+4=7; Grade group 2);	moderate systemic arteriosclerosis
12	15	8	-weight: 1860g<br>-DAD proliferative phase in all lobes<br>-focal acute bronchopneumonia<br>(microbiology: *serratia marcescens*)<br>-pleural effusion: absent	weight 540g; biventricular hypertrophy; focal amyloidosis; CAD with stenosis up to 50% and two scar regions (2cm each) and aneurysm; pacemaker in place;		signs of shock in kidney and liver; liver with central fibrosis (METAVIR F2); moderate systemic arteriosclerosis; focal renal infarction with a fresh thromb-embolus; femoral deep venous thrombosis;

DAD, diffuse alveolar damage; AFOP, acute fibrinous and organizing pneumonia; CAD, coronary artery disease;

Supplementary Table S3. Detailed histological pulmonary findings of the patient cohort.

Case	Lung findings (detailed)											
	**congestion**	**Interstitial edema**	**Hyaline membranes**	**Intraalveolar fibrinous exudates**	**Pneumocyte atypia**	**Intracellular giant cells**	**organization**	**Squamous cell metaplasia**	**Bronchitis or pneumonia**	**endothelialitis**	**(micro-) thrombi and infarcts**	
1	yes	no	yes	no	mild	no	no	no	no	no	no	
2	yes	yes	yes	no	yes	no	no	no	acute bronchitis with associated suppurative bronchopneumonia in all lobes (UL predominant)	yes	no	
3	yes	yes	yes	yes	yes	yes	no	yes	suppurative and necrotising bronchopneumonia in all lobes	yes	microthrombi, no infarcts	
4	yes	no	yes	no	yes	few	beginning	yes	minimal chronic bronchitis	yes	microthrombi, no infarcts	
5	yes	yes	yes	no	yes	no	no	yes	suppurative bronchopneumonia in all lobes; large bronchi without inflammation.	yes	microthrombi, no infarcts	
6	yes	yes	yes	yes (associated with pneumonia)	yes	yes	beginning	focal	focal acute bronchitis with associated suppurative (and focally necrotising) bronchopneumonia in all lobes	no	no	
7	yes	yes	no	present	no	no	no	no	suppurative and necrotising bronchopneumonia in all lobes with abundant bacteria.	no	no	

8	yes	yes	yes	yes	yes	few	yes	yes	mild chronic bronchitis	yes	peripheric emboli with focal associated hemorrhagic infarction	
9	yes	yes	yes	yes	yes	yes	beginning	focal	mild chronic bronchitis	yes	no	
10	yes	yes	no	yes	yes	yes	yes	yes	no	yes	no	
11	yes	yes	no	Focal, associated with pneumonia	severe	yes	yes	prominent	focal acute bronchitis in all lobes, with associated mucostasis and small foci of pneumonia; in the left superior lobe bronchitis due to non-invasive aspergilloma, with ulcus and peribronchial necrotising, absceding pneumonia and surrounding AFOP, and blood clots in the bronchial lumina	no	no	
12	present	present	present	present	present	present	present	no	focal pneumonia (right lower lobe)	yes	no	
